# What is OSFED? The predicament of classifying ‘other’ eating disorders

**DOI:** 10.1192/bjo.2021.985

**Published:** 2021-08-12

**Authors:** Zoe M. Jenkins, Serafino G. Mancuso, Andrea Phillipou, David J. Castle

**Affiliations:** Department of Mental Health, St. Vincent's Hospital, Melbourne, Australia, and Department of Psychiatry, The University of Melbourne, Melbourne, Australia; Department of Psychiatry, The University of Melbourne, Melbourne, Australia; Department of Mental Health, St. Vincent's Hospital, Melbourne, Australia, Department of Psychiatry, The University of Melbourne, Melbourne, Australia, and Centre for Mental Health, Swinburne University of Technology, Melbourne, Australia; Department of Mental Health, St. Vincent's Hospital, Melbourne, Australia, and Department of Psychiatry, The University of Melbourne, Melbourne, Australia

**Keywords:** Nosology, anorexia nervosa, eating disorders NOS, bulimia nervosa, binge eating disorder

## Abstract

The transition from DSM-IV to DSM-5 relaxed diagnostic criteria for anorexia nervosa and bulimia nervosa, and recognised a third eating disorder, binge eating disorder. However, a large proportion of cases remain in the ill-defined category of ‘other specified feeding and eating disorders’. We sought to investigate the utility of a proposed solution to classify this group further, subdividing based on the dominant clinical feature: binge eating/purging or restraint. Cluster analysis failed to identify clusters in a treatment-seeking sample based on symptoms of restraint, binge eating, purging and over-evaluation of shape and weight. Further investigation of this highly heterogeneous group is required.

The DSM-5^1^ specifies three main eating disorders: anorexia nervosa, bulimia nervosa and binge eating disorder (BED). People with eating disorders who do not meet full criteria for any of the above disorders fall into the nebulous categories ‘other specified feeding or eating disorder’ (OSFED) or ‘unspecified feeding or eating disorder’ (UFED). The OSFED category contains atypical anorexia nervosa, subthreshold bulimia nervosa and BED among others, whereas UFED is designated when full criteria for other eating disorders are not met or insufficient information is known.

Prior to the introduction of the DSM-5, Fairburn and Cooper^2^ proposed three solutions in order to classify and understand these groups further: (a) relax the diagnostic criteria for anorexia nervosa and bulimia nervosa and recognise BED as a third eating disorder in the DSM-5; (b) reclassify the OSFED/UFED group as an additional eating disorder termed ‘mixed eating disorder’; and (c) subdivide the group on the basis of the dominant clinical feature, *viz.*: *purge* (recurrent self-induced vomiting, laxative misuse or overexercising, often accompanied by subjective binge eating) and *restraint* (extreme dietary restraint and low body mass index (BMI)). It was also suggested that most have over-evaluation of shape and weight, and that the extent to which their weight varies is dependent on the patient's dominant clinical feature.^2^ The first proposal was successfully adopted by the DSM-5, and although DSM-5 provides a better accommodation of the complexities of the range of eating disorders than DSM-IV, OSFED/UFED still subsumes a substantial proportion of patients with disordered eating.^3^ More troubling is the heterogeneity of the OSFED/UFED group, which poses a challenge for clinicians regarding treatment options and prognostic outcomes.^2^ In light of this, we returned to the third model proposed by Fairburn and Cooper as it has clinical parsimony and sought to influence DSM-5. Rather than an appraisal of the entirety of the DSM-5 range of eating disorder diagnoses, we were interested specifically in the OSFED/UFED categories, which remain ill-defined. The current study aimed to interrogate Fairburn and Cooper's aforementioned third proposal in a large group of individuals with eating disorders. Accordingly, it was hypothesised that the OSFED/UFED group would subdivide into two groups based on dominant feature.

## Method

Data were from 390 patients attending the Body Image and Eating Disorders Treatment and Recovery Service at St Vincent's Hospital Melbourne, Australia, and are described elsewhere.^4^

### Diagnostic analysis

Algorithms (Supplementary Table 1 available at https://doi.org/10.1192/bjo.2021.985) were derived from DSM-5 criteria using scores on items from the Eating Disorder Examination-Questionnaire (EDE-Q) to classify individuals into one of four diagnostic groups:^3^ anorexia nervosa, bulimia nervosa, BED and OSFED/UFED (hereafter referred to as OSFED). The EDE-Q is a 28-item self-report measure that assesses the cognitive and behavioural symptoms of eating disorders.^5^ Respondents report on items in relation to the past 28 days, with higher scores indicating more severe symptoms or higher frequency.^6^

### Cluster analysis

Cluster analyses were performed using R 3.6.3 on the aforementioned OSFED group. Hierarchical Density-Based Clustering of Applications with Noise (HDBSCAN)^7^ was used to group patients based on their BMI, binge eating (EDE-Q items 13–15), purging (EDE-Q items 16–18), restraint (EDE-Q items 1–5) and over-evaluation (EDE-Q items 22, 23, 25, 26). Patients were excluded from the analysis if they had missing EDE-Q data on these items; as a result, the cluster analysis was performed on data from 104 OSFED participants (78.8%).

HDBSCAN generates a complete density-based clustering hierarchy from which only the most significant clusters are extracted. It also detects outliers. The HDBSCAN has a single input parameter, m_pts_, which can be interpreted as the minimum number of points close enough to each other to be considered a cluster and not an outlier.^8^ The optimal value for this parameter, the m_pts_ value with the highest average Silhouette index,^9^ was selected for further analysis using clValid.^10^ The Silhouette index is an internal validation index that measures the degree of confidence in the clustering assignment of an observation.

The above steps were performed on normalised and scaled data (i.e., *Z*-scores) given that the variables of interest were measured on different scales.

### Cluster comparisons

Comparisons were performed using SPSS (IBM, SPSS Statistics version 25). To compare the clusters on specific EDE-Q items, independent *t-*tests were performed; significance was set at *P* < 0.05. The Bonferroni–Holm *P*-value correction for multiple testing was used to keep the family-wise error rate at 5%.

### Ethics approval and consent to participate

The study was granted ethics approval from the Human Research Ethics Committee at St Vincent's Hospital, Melbourne and all procedures were in line with the Declaration of Helsinki. Informed consent was obtained from all participants.

## Results

The 390 participants included in the study comprised 367 (94.1%) females, with an age range of 18–78 years (M = 27.2(9.91)); BMI ranged from 10.90 to 47.70 (M = 18.71(4.45)). The most prevalent diagnosis was anorexia nervosa (*N* = 158; 40.5%), with 132 (33.8%) receiving a diagnosis of OSFED, 87 (22.3%) bulimia nervosa and 13 (3.3%) BED (see Supplementary Table 2 for descriptive characteristics).

**Fig. 1:**
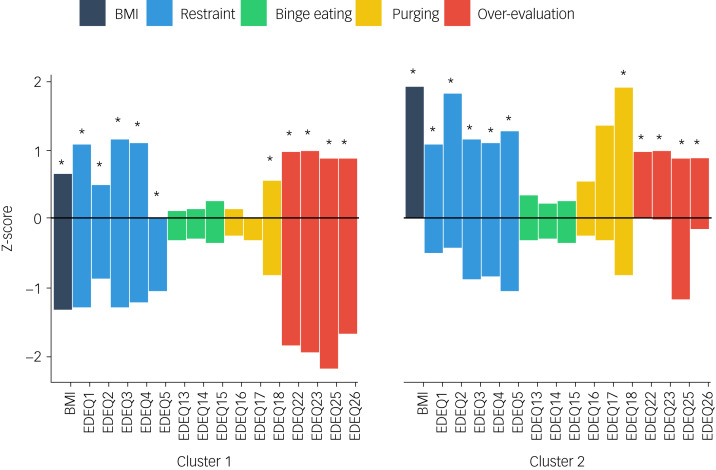
Cluster analysis of OSFED participants on BMI and EDE-Q items of restraint, binge eating, purging and overvaluation. * denotes significant difference on item between clusters, *P* < 0.001; BMI: body mass index; EDE-Q: eating disorder examination questionnaire; OSFED: other specified feeding or eating disorder.

An *m*_pts_ value of 6 was found to have the highest average Silhouette index (Supplementary Table 3). Using this parameter value, two clusters of OSFED patients were found, comprising 25 (24%) participants in the first cluster and 25 (24%) in the second cluster (Fig. 1). Fifty-four OSFED participants (51.9%) were identified as outliers.

Individuals included in cluster 1 had significantly lower BMI than individuals included in cluster 2 (M ± s.d. of 16.36 ± 1.78 *v.* 20.37 ± 1.44, *P* < 0.001). Cluster 1 also had significantly lower scores than cluster 2 on the following individual EDE-Q items: all restraint items (*P* < 0.001), purge item 18 (*P* < 0.001), and all over-evaluation of shape and weight items (*P* < 0.001). Clusters did not significantly differ on scores of EDE-Q binge items 13, 14 or 15, or purge item 16 or 17 (see Supplementary Fig. 1 for a scatter plot of average EDE-Q restraint z-scores vs. average EDE-Q binge/purge z-scores).

## Discussion

The current study sought to test the proposal that individuals in the OSFED classification can be delineated into two groups, one characterised by binge/purge behaviours and the other by dietary restraint.^3^ While this hypothesis was not supported in the current sample, OSFED was the second most prevalent eating disorder diagnosis, reflective of previous findings.^11^ Cluster analysis on BMI and symptoms of restraint, binge, purge and over-evaluation revealed two distinct clusters of OSFED individuals. However, over half of the individuals who received an OSFED diagnosis were not included in either cluster, suggesting that symptoms of binge/purge and restraint, as assessed by the EDE-Q, failed to define two groups in the current sample. Further analysis of the cluster features revealed that one cluster was characterised by lower BMI and *decreased* eating disorder behaviours of restraint, binge, purge, and over-evaluation of weight and shape. This may suggest that other behavioural equivalents, including developmental stage and culture-specific manifestations, are better indicators of eating disorder psychopathology. Moreover, given evidence of frequent diagnostic transitions and presentation changes,^1^ there may be significant clinical utility in routine assessment of past eating disorder symptomatology for treatment and prognosis in those who receive a vague diagnosis of OSFED/UFED. Although the primary objective of diagnostic classification is to inform treatment, it is pertinent that schemes are informed by both clinical and epidemiological research. A tentative suggestion, in line with Fairburn and Cooper's remaining proposed solution to reclassify the OSFED group as an additional eating disorder termed ‘mixed eating disorder’, is consideration of a combined eating disorder diagnostic group that incorporates sequential diagnoses and presentations. However, further research is required to confirm any hypotheses and improve clinical utility through understanding of the OSFED group.

Generalisations from the current study are limited by the use of EDE-Q algorithms to determine eating disorder diagnosis and the use of a treatment-seeking sample, who may not represent the wider community of people with disordered eating.

The current study failed to identify specific symptom clusters that represented the OSFED group as a whole, based on BMI and symptoms of restraint, binge eating, purging, and over-evaluation of shape and weight. It highlighted that some individuals do not endorse eating disorder behaviours as currently assessed by the DSM-5 (namely binge/purge or restraint) yet are significantly impaired in physical health or psychosocial functioning and present for treatment. Further investigation into the clinical characteristics of this proposed eating disorder, and associated eating disorder behaviours, is required given that that OSFED includes a highly heterogeneous group of individuals that remain difficult to incorporate into the diagnostic classifications as they currently stand.

## Data Availability

The data that support the findings of this study are available from the corresponding author, Z.M.J., upon reasonable request.

## Supplementary material

For supplementary material accompanying this paper visit http://dx.doi.org/10.1192/bjo.2021.985. click here to view supplementary material
