# Supplementary material for: What is OSFED? The predicament of classifying ‘other’ eating disorders
Source: BJPsych Open. 2021 Aug 12;7(5):e147. doi: 10.1192/bjo.2021.985 (PMC8388009; doi:10.1192/bjo.2021.985)
Supplement: Supplementary file 1 [file S2056472421009856sup001.zip › Supplementary_Material_3.docx]

**Supplementary Material 3**

Table S3. Number of clusters and mean cluster stability and membership probability scores for different m_pts_ values

| ***m*_pts_** | **Clusters** | **Stability Score** | **Membership Probability** |
| --- | --- | --- | --- |
| 3 | 6 | 1.586 | 0.149 |
| 4 | 4 | 1.732 | 0.135 |
| 5 | 3 | 1.714 | 0.142 |
| **6** | **2** | **2.524** | **0.158** |
| 7 | 2 | 1.893 | 0.142 |
| 8 | 2 | 1.660 | 0.130 |
| 9 | 2 | 1.313 | 0.114 |
| 10 | 2 | 1.094 | 0.106 |
| 11 | 2 | 0.666 | 0.079 |
| 12 | 2 | 0.677 | 0.081 |
| 13 | 2 | 0.685 | 0.082 |
| 14 | 2 | 0.641 | 0.085 |
| 15 | 2 | 0.530 | 0.077 |
