# Supplementary material for: What is OSFED? The predicament of classifying ‘other’ eating disorders
Source: BJPsych Open. 2021 Aug 12;7(5):e147. doi: 10.1192/bjo.2021.985 (PMC8388009; doi:10.1192/bjo.2021.985)
Supplement: Supplementary file 1 [file S2056472421009856sup001.zip › Supplementary_Material_2.docx]

**Supplementary Material 2.**

Table S2. Demographic characteristics of sample

| **Characteristic** | ***N*(%) or M±SD** |
| --- | --- |
| Total | 390 (100.0%) |
| Age at assessment (years) | 27.16±9.90 |
| Gender |  |
| Male | 23 (5.9%) |
| Female | 367 (94.1%) |
| Age at ED onset (years) | 17.48±6.51 |
| Duration of illness (years) | 9.10±8.98 |
| Ethnicity |  |
| Aboriginal and Torres Strait Islander | 6 (1.5%) |
| Caucasian | 304 (77.9%) |
| East Asian | 13 (3.3%) |
| Middle Eastern | 1 (0.3%) |
| African | 4 (1.0%) |
| Other European | 24 (6.2%) |
| Other | 12 (3.1%) |
| Missing | 26 (6.7%) |
| Employment |  |
| Part-time | 49 (12.6%) |
| Full-time | 72 (18.5%) |
| Student | 136 (34.9%) |
| Home duties | 13 (3.3%) |
| Unable to work due to illness | 72 (18.5%) |
| Unemployed | 34 (8.7%) |
| Missing | 14 (3.6%) |
| Education |  |
| Primary school | 1 (0.3%) |
| Secondary school | 102 (26.2%) |
| Tertiary commenced | 137 (35.1%) |
| Tertiary completed | 105 (26.9%) |
| Vocational | 12 (3.1%) |
| Missing | 33 (8.5%) |
