# Supplementary material for: What is OSFED? The predicament of classifying ‘other’ eating disorders
Source: BJPsych Open. 2021 Aug 12;7(5):e147. doi: 10.1192/bjo.2021.985 (PMC8388009; doi:10.1192/bjo.2021.985)
Supplement: Supplementary file 1 [file S2056472421009856sup001.zip › Supplementary_Material_1.docx]

**Supplementary Material 1**

Table S1. EDE-Q algorithm used to derive eating disorder diagnoses

| **Criteria** | **Description** | **EDE-Q item** |
| --- | --- | --- |
| **Anorexia nervosa** |  |  |
| A.  Low weight | BMI < 18.5 | Clinical measurement |
| B.  Fear of weight gain | Fear of weight gain | Item 10 ≥ 4 |
| C.  Body image disturbance | Importance of weight OR importance of shape | Item 22 ≥ 4 OR Item 23 ≥ 4 |
| AN Diagnosis | All satisfied | A & B & C |
| **Bulimia nervosa** |  |  |
| A.  Binge eating | OBEs endorsed | Item 15 ≥ 4 |
| B.  Compensatory behaviour | Vomiting, laxative use, or overexercise endorsed | Items 16+17+18 ≥ 4 |
| C.  Frequency/duration | Total OBEs ≥ once per week and total compensatory behaviour ≥ once per week | Items 16+17+18 ≥ 4 |
| D.  Overvaluation of shape and weight | Importance of weight OR importance of shape | Item 22 ≥ 4 OR Item 23 ≥ 4 |
| E.  Not AN | DSM-5 criteria for AN not met |  |
| BN diagnosis | All satisfied | A & B & C & D & E |
| **Binge eating disorder** |  |  |
| A.  Binge eating | OBEs endorsed | Item 15 ≥ 4 |
| B.  Frequency/duration | Total OBEs ≥ once per week | Item 15 ≥ 4 |
| C.  No regular compensatory behaviours, not BN, not AN | Total compensatory behaviour < 4 episodes per month and DSM-5 criteria for AN and BN not met | Items 16+17+18 < 4 |
| BED diagnosis | All satisfied | A & B & C |
| **OSFED/UFED** |  |  |
| A.   Clinically significant eating disorder that does not meet criteria for AN, BN, or BED | DSM-5 criteria for AN, BN, or BED not met |  |
