# Supplementary figures and images for: What is OSFED? The predicament of classifying ‘other’ eating disorders
Source: BJPsych Open. 2021 Aug 12;7(5):e147. doi: 10.1192/bjo.2021.985 (PMC8388009; doi:10.1192/bjo.2021.985)

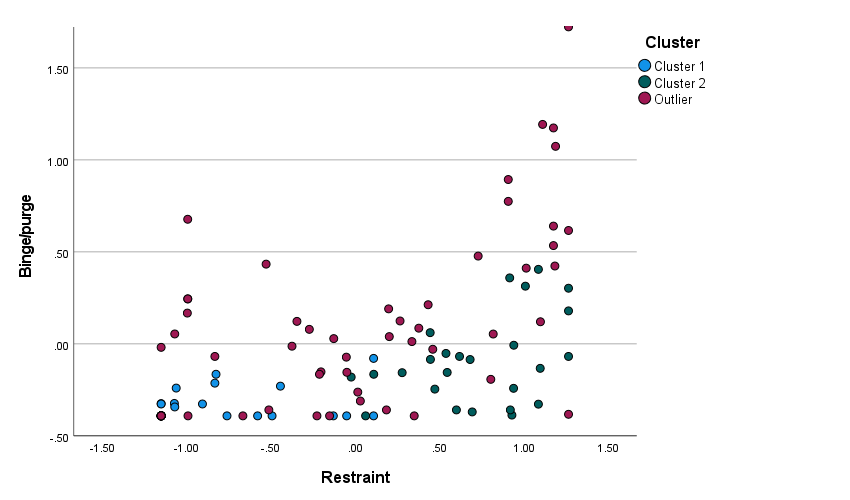


Figure S2. Scatter plot of average EDE-Q restraint z-scores vs. average EDE-Q binge/purge z-scores.

Supplement: Supplementary file 1 [file S2056472421009856sup001.zip › Figure_S2.docx]
